# The Sick in Scottish Prisons

**Published:** 1897-01-23

**Authors:** 


### THE SICK IN SCOTTISH PRISONS.

A letter recently addressed by the secretary of the Howard Association to the editor of the *Times* contained a strong statement as to certain reforms which clearly seem to be urgently necessary in the prisons of Scotland. Some months ago a deputation, composed of the chief officers of those establishments, waited on Lord Balfour of Burleigh to urge upon the Government the importance of at least two prominent changes in the interior arrangements of the gaols to which they belonged. The first of these was the raising of the salaries of the governors, medical officers, chaplains, and others to a level with the remuneration paid to similar functionaries in English and Irish prisons. The second was the necessity of a considerable increase in the inadequate staff of warders, especially in view of the very objectionable association of weak-minded prisoners with other criminals, and that without due supervision, which is involved in the present condition of affairs. With the first of these requirements—the increase of salaries—though perfectly just, we have nothing to do; but the second is a matter of vital importance affecting not only members of the medical profession, but also all those who in any capacity desire to promote the welfare of their fellow-creatures. It is astonishing that the discreditable state of matters described in this letter, should have been allowed to exist so long; but perhaps those who have no opportunity of scrutinising the interior life of prisons, may hardly be aware of the serious evils involved in it. The description given of the existing arrangements is really somewhat appalling, as may be seen by the following statements given by the *Times* correspondent: "At present in some of the Scottish gaols, not only are the warders and other officers too few in number to take te o versight of special cases in the cells, but even to look after the hospital patients. Thus, in one prison lately there were in the hospital four invalids, of whom one was suffering from bleeding at the lungs, one from epilepsy, and two others from wounds—these shut up all night with another prisoner to look after them! What do the authorities think of the nursing first, and secondly of the association in such a case? A murder might easily have been committed under the circumstances." It is a fact that murders have resulted from the indiscriminate mingling together of infirm prisoners of the lowest class and the most unsatisfactory character in the sick ward, some of them not being even in full possession of their senses. We have ample testimony in the records of workouses and lunatic asylums that the principle is radically wrong, which allows unreliable and uninstructed persons to be put in charge of others of their class, even when they are not physically or mentally afflicted; but that so thoroughly unsound a system should be permitted in the prison infirmaries of Scotland seems almost incredible. The intercourse of prisoners with each other in any shape or way ought to be as strictly prohibited in Scotland as it is in the English and Irish gaols. But when we come to look on such an arrangement for the nursing and care of criminals whose errors have separated them from family and friends, to cast them wholly on the responsibility of the State, it is not easy to measure the condemnation due to such unseemly proceedings. An order emanating from the Home Office some time since put a final stop to anything of the kind in English gaols; but irregularities in Scottish prisons appear to have escaped the notice of their own Commissioners. In our opinion, founded on actual experience, no prisoner ought ever to be alone with another under any circumstances, and even the slight deviation from the rules enforcing the separate system, which allows a prisoner to assist an officer in the care of persons subject to fits or to temporary insanity, is not without its difficulties; while the Scottish practice of placing patients under the care of a companion criminal is completely indefensible. The proper treatment of the sick who, by their own misdeeds, have been relegated to the charge of the Government, is of such weighty importance that a certain number of warders, both male and female, ought to be trained in skilful and intelligent nursing as part of their permanent duties, and we hope to see the day when this plan will be carried out, and a section of the regular prison staff be suitably prepared for infirmary duties. In the meantime, the condition of hospital arrangements in the Scottish prisons revealed by the secretary of the Howard Association requires the most speedy and decided reform. If this can only be obtained by a large addition to the staff of officers and an increase of their salaries, it is to be hoped that no motives of economy will stand in the way of the prompt adoption of measures on behalf of our fellow-creatures—criminal though they be—which justice and mercy alike demand at the hands of the State.

